# Detection of rare autoreactive T cell subsets in patients with pemphigus vulgaris

**DOI:** 10.3389/fimmu.2022.979277

**Published:** 2022-09-20

**Authors:** Alexandra Polakova, Leonie Kauter, Adina Ismagambetova, Dario Didona, Farzan Solimani, Kamran Ghoreschi, Michael Hertl, Christian Möbs, Christoph Hudemann

**Affiliations:** ^1^ Department of Dermatology and Allergology, Philipps-Universität Marburg, Marburg, Germany; ^2^ Department of Dermatology, Venereology and Allergology, Charité-Universitätsmedizin Berlin, Berlin, Germany; ^3^ Berlin Institute of Health at Charité – Universitätsmedizin Berlin, BIH Biomedical Innovation Academy, Berlin, Germany

**Keywords:** CD154, CD40L, CD4+ T cells, pemphigus, autoreactive, desmoglein, autoimmunity

## Abstract

Analysis of T lymphocyte proliferation and activation after antigenic or mitogenic stimulation is a vital parameter used in the diagnosis of various immuno-deficiencies and during the monitoring of treatment responses. Most applied techniques are based on the incorporation of tritiated thymidine (^3^H-TdR) or ELISPOT analysis, both rely on rather time-consuming/-intensive *ex vivo* protocols or encompass inherent drawbacks such as the inability to distinguish specific cell populations (^3^H-TdR, ELISPOT) or focus on a single cytokine (ELISPOT). Here we aimed at characterizing the rapid expression of intracellular CD154 (CD40L) as a marker for rare antigen-specific CD4+ T cells in pemphigus vulgaris (PV). Upon stimulation with human desmoglein (Dsg) 3, the major autoantigen in PV, the expression of CD154 was significantly increased in PV patients compared to healthy controls (HC) and correlated with anti-Dsg3 IgG titers. Patients with active disease showed higher numbers of Dsg3-reactive CD4+ T cells in CXCR5+ T follicular helper cells. In remittent PV and HC, CXCR5+CD4+ T cells remained largely unaffected by Dsg3. IL-17 and IL-21 expression were significantly induced only in CD154+CD4+ T cells from PV patients, lending themselves as potential novel treatment targets. Additionally, stimulation with immunodominant Dsg3-derived epitopes strongly induced a CD4+ T cell response *via* CD40-CD154 interaction similar to the human Dsg3 protein. We here established a rapid *ex vivo* assay allowing the detection of Dsg3-reactive CD4+ T cells from activated systemically available PBMCs, which further supports the crucial concept of antigen-specific T cells in the pathogenesis of PV.

## Introduction

Pemphigus vulgaris (PV) is a rare and potentially life-threatening autoimmune disease, characterized by a production of autoantibodies targeting desmosomal proteins, namely desmoglein (Dsg)3 and/or Dsg1 (1). Binding results in a loss of adhesion between keratinocytes (referred to as acantholysis), leading to the occurrence of intraepidermal blisters and erosions of skin and/or mucous membranes (2). Different expression patterns of Dsg3 and Dsg1 give rise to either the mucosal-dominant PV (Dsg3) or the primarily skin-associated pemphigus foliaceus (PF; Dsg1) (1, 3). Pathogenic anti-Dsg3 and -Dsg1 IgG autoantibodies recognize both N- and COOH-terminal epitopes of the human Dsg3 ectodomain and are widely known to play a crucial role in the manifestation of PV (4, 5). Autoantibody formation originates in the activation of Dsg3-reactive CD4+ T cells by Dsg3 peptides binding to HLA class II and *via* CD40-CD40L-dependent T cell-B cell interaction. This Th2/Th17 driven process is critical for the induction and maintenance of autoreactive memory B cells as precursors of autoantibody-producing plasma cells (6–9). It is widely regarded that the HLA class II alleles HLA-DRB1*04:02 and HLA-DQB1*05:03 confer susceptibility to PV (10) and Dsg3-reactive CD4+ T cell responses against the ectodomain of Dsg3 were identified in PV patients (11). So far, several Dsg3 peptides were identified as potential immunodominant Dsg3 epitopes recognized by CD4+ T cells, which in the context of the PV-associated HLA-alleles, were shown to induce the formation of Dsg3-specific IgG in a humanized HLA-transgenic mouse model (12).

Analysis of T lymphocyte proliferative responses to antigenic or mitogenic stimuli is a vital parameter used in both diagnosis and monitoring of a variety of immune responses. Most commonly applied techniques are based on the incorporation of tritiated thymidine (^3^H-TdR) or enzyme-linked immunospot (ELISPOT) analysis (13, 14). Both rely on a rather long *ex vivo* expansion periods and stimulation protocols (up to 11 days). Additionally, these methods contain inherent drawbacks such as the inability to distinguish specific cell populations (3H-TdR, ELISPOT) using peripheral blood cells as well as the assumption of the importance of certain cytokines (ELISPOT) (15–17). Rapid identification and quantification of rare antigen-specific T cell numbers as a marker for treatment-response is of importance in diseases such as PV (18). To overcome this obstacle, here we introduce a concept for the assessment of antigen-specific CD4+ T cells based on CD154 expression after short-term *ex vivo* stimulation with defined antigens or epitopes. CD154 (CD40 ligand; CD40L) is an activation-induced T cell surface glycoprotein belonging to the tumor necrosis factor receptor (TNFR) superfamily. In T cell-mediated immune responses and inflammation, interactions between CD154 and CD40 provide essential signals in T cell priming and T cell effector functions and subsequently induce B cell proliferation and differentiation, isotype-switching, and formation of memory B cells (19–21). CD154 is transiently expressed on activated, but not resting CD4+ T cells, therefore, it cannot be used in long-term assays for the analysis of antigen-specific CD4+ T cells (20). Its expression is directly correlated to CD4+ T cells specific for defined antigens in several diseases such as pathogenic infection, viral immunity, and self-tolerance (22–24). Its increased expression was observed as a marker of disease activity in rheumatoid arthritis, multiple sclerosis, and systemic lupus erythematosus (25–27). Moreover, in a pemphigus mouse model, administration of anti-CD154 mAb blocked anti-Dsg3 IgG production and prevented blister formation (28). The latter indicates the quintessence of CD40-CD154 interaction for the induction of pathogenic IgG anti-Dsg3 antibodies and, when blocked, induction of tolerance.

In this study, we sought to analyze the presence of antigen-specific CD4+ T cells with regards to an activation marker CD154 combined with previously suggested cytokine analysis in PV patients using multicolor flow cytometry. By using full-length human Dsg3 or Dsg3-specific epitopes for *ex vivo* stimulation, we show that expression of the activating CD154 of PBMCs from PV patients can be found in CXCR5+ T follicular helper cells associated with IL-21 expression, further supporting the concept that antigen-specific T cells are central for the pathogenesis of PV. These findings underline the concept that activating T cell responses against Dsg3 may be critical in driving the IgG-dependent immune pathogenesis and identify IL-21 as a potential target in pemphigus.

## Materials and methods

### Study participants

A total of 33 patients with PV were included in this study by the Department of Dermatology and Allergology at the University Hospital Giessen and Marburg and the Department of Dermatology, Venereology and Allergology at the Charité–Universitätsmedizin Berlin (**Table 1**). This study was approved by the Ethics Committees of the Medical Faculty of the Philipps-Universität Marburg (Az. 20/14) and the Charité Berlin (AZ: EA4/194/19) and was conducted according to the principles of the Declaration of Helsinki. Written informed consent was obtained from all participants. Inclusion of all tested subjects, apart from clinical presentation of erosions and flaccid blisters on the skin and/or mucous membrane, was made using at least one of the following diagnostic criteria: serological analysis of titers of circulating anti-Dsg1 and anti-Dsg3 IgG autoantibodies, histopathological evidence of intraepidermal acantholysis and/or epidermal intracellular deposition of IgG and/or complement factor C3 using direct immunofluorescence of perilesional skin biopsy and/or indirect immunofluorescence using monkey esophagus. To distinguish mucocutaneous from the mucosal-dominant pemphigus phenotype, serological autoantibody titers were analyzed in serum using both the commercially available anti-Dsg1 and anti-Dsg3-ELISA (Euroimmun, Lübeck, Germany). PV patients were further classified into disease stages of relapse (active) and remission with respect to their clinical disease activity and systemic therapy (29). Epidemiological data, clinical status, therapy and Dsg1 or 3 titers at the time of analysis are shown in the **Supplementary Table 1**.

**Table 1:** Characteristics of pemphigus patients and HC.

		Individuals
**Healthy control group size**	N	23
**Demographics**	Age, median (range)	33 (22-58)
	Male (n, %)	15 (65%)
	Female (n, %)	8 (35%)
**Pemphigus patients group size**	N	33
**Demographics**	Age, median (range)	60 (41-83)
	Male (n, %)	16 (48%)
	Female (n, %)	17 (52%)
**Clinical status**	Active	9 (27%)
	Remission	24 (73%)
**Clinical phenotype**	Mucosal	9 (30%)
	Cutaneous	4 (13%)
	Mucocutaneous	8 (27%)
	None	9 (30%)
**Pemphigus specific HLA-types**	HR DRβ1*0402	5 (25%)
	HR DQβ1*0503	7 (35%)
	Both	5 (25%)
	None	3 (15%)

Demographics among PV patients include clinical status, clinical phenotype and pemphigus specific HLA-type.

Healthy controls (HC; n=23) were recruited at the Department of Dermatology and Allergology and had no history of autoimmune skin diseases (**Supplementary Table 2**).

### Human Dsg3 and peptides

The extracellular domain of human Dsg3 was produced in baculovirus-infected insect cells (High Five, Carlsbad, CA, USA) as described previously (30). Proteins were purified by affinity chromatography using nickel-nitrilotriacetic agarose beads (Quiagen, Hilden, Germany) according to the manufacturer’s instructions. Purified protein was gradually dialyzed against PBS supplemented with 0.5mmol/L CaCl_2_. Quality control was done by coomassie staining, ELISA with patients’ sera and western-blot analysis with Dsg3-specific antibodies. Fifteen-mer Dsg3 peptides used for *ex vivo* stimulation were synthesized by ProImmune (thinkpeptides, Oxford, UK; **Figure 3A**).

### Antigen stimulation

Human peripheral blood mononuclear cells (PBMCs) were isolated from citrate-phosphate-dextrose-adenine (CPDA)-containing peripheral blood samples by density gradient sedimentation using Lymphocyte Separation Medium (Capricorn Scientific, Ebsdorfergrund, Germany), washed twice in PBS and plated in round-bottom 96-well plates at a concentration of 3 x 10^5^ cells/200µl in a complete RPMI 1640 medium (Capricorn Scientific, Ebsdorfergrund, Germany) containing 10% fetal bovine serum (FBS; Sigma-Aldrich, St. Louis, MO). Freshly isolated cells were immediately stimulated after PBMC isolation. Thawed PBMCs were first rested for 22 hours at 37°C and 5% humidified CO_2_ to improve antigen-induced CD154 expression. Subsequently, PBMCs were stimulated with one of the following: recombinant human Dsg3_1-566_ (10-20 µg/ml), four Dsg3 epitopes (P1_190-204_, P2_206-220_, P3_251-265_, P4_375-391_; 10 µg/ml), PHA (phytohemagglutinin, 10 µg/ml, Sigma-Aldrich, St. Louis, MO) used as a positive control and unstimulated PBMCs served as a negative control. All samples were treated with Brefeldin A (10 µg/ml, Invitrogen by Thermo Fisher Scientific, Waltham, Massachusetts) 4 hours prior to analysis. Subsequently, cells were stained for flow cytometry as described below.

### Flow cytometry staining and data analysis

For flow cytometry, cells were coated with Fc-block (10 μl of 1: 100 diluted TruStain Fc block, Biolegend, San Diego, CA) for 10 minutes and then stained with fluorescently labeled antibodies for 30 minutes at room temperature. For extracellular staining, the following monoclonal antibodies were used: CD4-AF 700 (RPA-T4, BD Biosciences, San Jose, CA, USA), CD8-PE-Cy7 (SK1), CXCR3-BV421 (G025H7), CD4-BV510 (RPA-T4), CD45RA-FITC (HI100), CD3-PerCP-Cy5.5 (SK7), CXCR5-PE (J252D4), CCR6-APC (G034E3; all BioLegend). For intracellular staining, cells were fixed and permeabilized using fixation/permeabilization buffer (eBioscience FOXP3/Transcription; Invitrogen, San Diego, CA) and stained intracellularly for 20 minutes at 4°C for functional readouts in permwash buffer (eBioscience FOXP3/Transcription; Invitrogen, San Diego, CA). For intracellular staining, the following monoclonal antibodies were used: CD154-BV421 (24-31), CD154-PE/Cyanine7 (24-31; both BioLegend), IL-4-BV786 (MP4-25D2), IFN-γ-FITC (B27), IL-17A-BV650 (N49-653), IL-21-PE (3A3-N2.1; all BD Biosciences, San Jose, CA, USA). Samples were acquired on the BD FACS LSR Fortessa equipped with four lasers (BD Biosciences, San Jose, CA). Standard flow cytometry data analysis was performed using BD FACSDiva Software (BD Biosciences, San Jose, CA, USA) and FlowJo version 10.8 (BD Biosciences, San Jose, CA).

### Statistical analysis

Statistical analysis was conducted using Graph Pad Prism version 8 software (GraphPad Software, La Jolla, CA). Statistical significance was calculated using a nonparametric, two-tailed unpaired Mann-Whitney U test and for multiple comparisons Kruskal-Wallis test followed by Dunn’s multiple comparisons; *p<0.05, **p<0.01, ***p<0.001.

## Results

### Kinetic *ex vivo* analysis of CD154 expression

**Figure 1:**
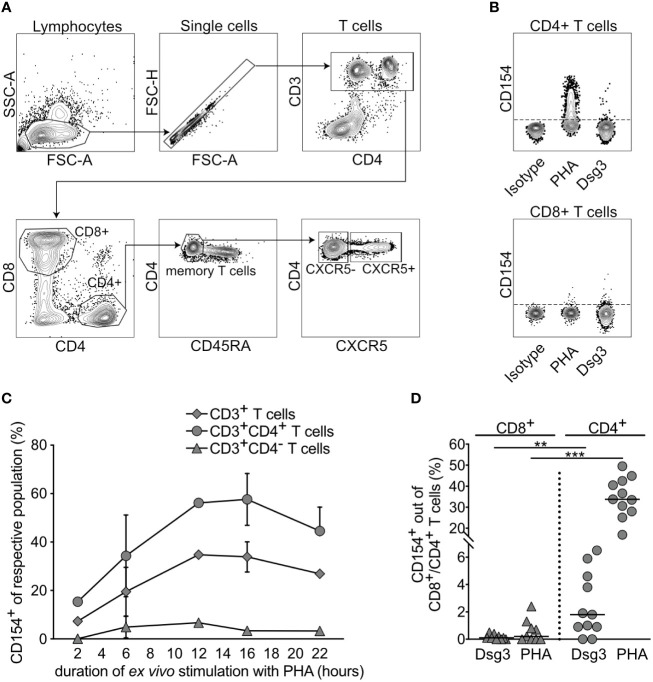
CD154 expression of CD3+ T cells and their subpopulations upon *ex vivo* stimulation. **(A)** Gating strategy to identify lymphocytes, single cells, T cells in peripheral blood, CD4+ and CD8+ T cells, memory T cells, CXCR5- and CXCR5+CD4+ T cells. FSC-A, forward scatter area; SSC-A, side scatter area; FSC-H, forward scatter height. **(B)** Representative flow cytometry plots portraying CD154 expression by CD4+ T cells upon antigenic (Dsg3) and mitogenic (PHA) *ex vivo* stimulation for 16 hours. **(C)** Kinetic analysis of relative CD154 frequency, identified by flow cytometry in peripheral blood, upon polyclonal stimulation out of respective parent population, i.e. CD3+ T cells, CD3+CD4+ T cells, CD3+CD4- T cells, CD3+CD4+CXCR5- T helper and CD3+CD4+CXCR5+T follicular helper cells, respectively. (n=1-3). **(D)** Relative frequency of CD154 expression on CD8+ and CD4+ T cells in peripheral blood of PV patients upon specific (Dsg3) or nonspecific (PHA) *ex vivo* stimulation for 16 hours (n=9-11). p<0.01 **, p<0.001 ***.

To establish optimal conditions for high CD154 expression, PBMCs were polyclonally stimulated *ex vivo* with PHA for 2, 6, 12, 16 and 22 hours. Using flow cytometry, relative frequencies of CD154-expressing cells out of the respective parent population were identified and followingly 5 major cell types were analyzed, namely: (1) CD3+ T cells, (2) CD3+CD4- T cells, (3) CD3+CD4+ T cells. To determine different CD4+ T cell subsets, we further stratified our gating strategy based on the surface expression of the CXCR5 chemokine that mediates T cell mobilization and homing into secondary lymphoid organs (31) (**Figures 1A, B**). This allows discrimination between (4) CD3+CD4+CXCR5- T helper (Th) and (5) CD3+CD4+CXCR5+ T follicular helper (Tfh) cells. Fresh PBMCs were, as compared to previous storage in liquid nitrogen, more responsive in their ability to produce CD154 expression (**Supplementary Figure 1**). With prolonged periods of *ex vivo* stimulation, an increasing CD154 expression up to 16 hours in CD4+ T cells was observed (**Figure 1C**), however, after 16 hours a notable decrease of CD154 expression with a decrease of viable cells was found (not shown). Based on these results, a 16-hour stimulation period of PBMCs was further applied. Analysis of CD8+ and CD4+ T cell subpopulations in view of their ability to express CD154 after specific (Dsg3) and nonspecific (PHA) stimulation showed that even though CD8+ T cells express CD154, it remained unchanged after stimulation (**Figure 1D**). In contrast, CD4+ T cells display a significant increase in CD154 expression after both antigen-specific (Dsg3) and polyclonal (PHA) stimulation (**Figure 1D**). In summary, a 16-hour stimulation interval of fresh PBMCs was found optimal for evaluation of CD154-expressing T cell responsiveness to stimuli.

### CD154 marker allows for detection of autoreactive T cells in patients with pemphigus vulgaris

Next, a total of 25 PV patients and 22 HC (**Supplementary Table 1**) were further analyzed by applying the previously established optimal readout conditions. Patients were stratified into acute and remittent disease stage based on ABSIS score and appearance of new clinical lesions for ≥ 3 month (29). Polyclonal T cell reactivity with PHA was used as positive control for each subject. To calculate changes in CD154 expression upon Dsg3 stimulation within the CD4+ T cells, relative frequencies of CD154 expression by unstimulated PBMCs were subtracted from the stimulated samples.

**Figure 2:**
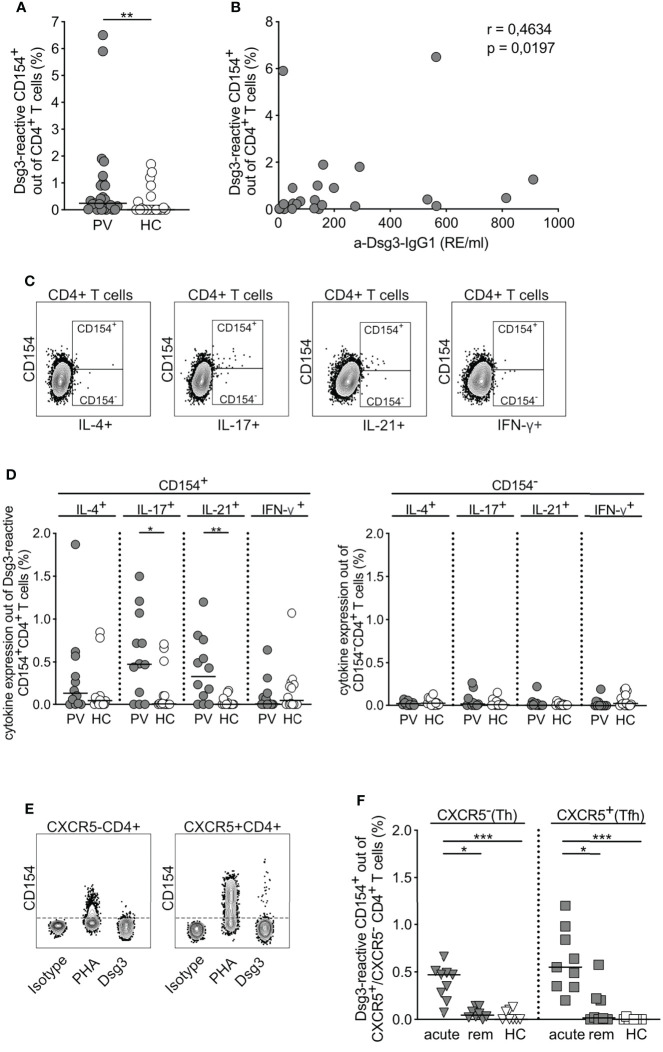
Characteristics of Dsg3-reactive CD154+ CD4+ T cells. **(A)** Relative frequency of CD154 expression upon *ex vivo* stimulation with Dsg3 in PV vs HC (PV n=25; HC n=22). **(B)** Correlation of anti-Dsg3-IgG1 serum concentration with relative frequencies of Dsg3-reactive CD154+CD4+ T cells in PV patients (n=25). Spearman’s rank correlation coefficient r=0.4634 and p=0.0197. **(C)** Representative flow cytometry plots depicting CD154- and CD154+ CD4+ T cells upon specific Dsg3 stimulation and their respective cytokine secretion of IL-4, IL-17, IL-21 and IFN-γ. **(D)** Relative frequency of IL-4, IL-17, IL-21 or IFN-γ cytokine expression in Dsg3-reactive CD154+CD4+ T cells on the left and CD154-CD4+ T cells (right; PV n=12; HC n=14). **(E)** Representative flow cytometry plots depicting CXCR5 expression in CD4+ T cells upon respective stimulation. **(F)** Relative frequencies of CD154 on CXCR5-CD4+ T (Th) cells and CXCR5+CD4+ T (Tfh) cells for acute PV, remittent PV patients and HC upon specific *ex vivo* stimulation with Dsg3 (acute PV, n=9; remittent PV, n=8; HC, n=8). **(D)** p<0.05 *, p<0.01 **, p<0.001 ***.

Stimulation with Dsg3 significantly increased CD154 expression in PBMCs derived from PV patients compared to HC (**Figure 2A**). Moreover, expression of CD154 on CD4+ T cells correlated positively with Dsg3 titers in pemphigus patients as shown by Spearman rank correlation (correlation coefficient 0.4634, p= 0.0197; **Figure 2B**). CD154+CD4+ T cells derived from PV patients were expressing significantly higher levels of IL-17 and IL-21 upon Dsg3 stimulation in contrast to HC and CD154-CD4+ T cells (**Figures 2C, D**). To further characterize CD4+ T cell subpopulations, the chemokine receptor CXCR5 was introduced, dividing CD4+ T cells into CXCR5-CD4+ Th and CXCR5+CD4+ Tfh cells (**Figure 2E**). Antigen-specific stimulation with Dsg3 resulted in a significant increase in CD154 expression on Th and Tfh cells alike, however, particularly in acute PV patients compared to HC and to a lesser extent in patients in clinical remission (**Figure 2F**). Taken together, CD154 marker expression associates with IL-17 and IL-21 expression specifically in PV patients, CXCR5+ Tfh cells react significantly upon Dsg3 stimulation allowing the detection of Dsg3-reactive CD4+ T cells in patients with PV.

### Dsg3-derived peptide P2 stimulated CD4+ T cells comparably with Dsg3

**Figure 3:**
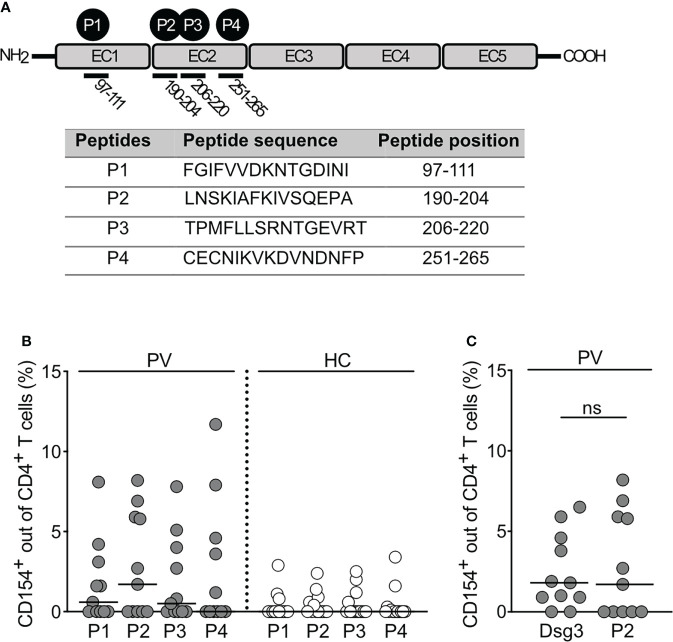
Induction of CD154 expression by Dsg3-specific T cell epitopes. **(A)** Summary of Dsg3-specific epitopes used for *ex vivo* stimulation. **(B)** Relative frequencies of CD154 expression in CD4+ T cells upon *ex vivo* stimulation with Dsg3-derived peptides (P1, P2, P3, P4) (PV n=11; HC n=14). **(C)** Relative frequency of CD154 expression out of CD4+ T cells upon *ex vivo* stimulation with Dsg3-derived peptide P2 and Dsg3 in PV patients (PV n=11).

The stimulatory effects using full protein might mask epitopes that are not recognized in a 3-dimensional structure. Besides the antigen-specific *ex vivo* stimulation with the full length Dsg3 and respective unstimulated control, we therefore analyzed the effects of a set of distinct Dsg3 epitopes for *ex vivo* stimulation. They all share a positively charged arginine (R) or lysine (K) at position 4 and are known to induce a proliferative *in vitro* response of peripheral T cells from PV patients. They are originally based on a peptide-binding algorithm for HLA-DRB1* 04:02, which shares similar binding motifs to HLA-DQB1*05:03 (13). Human Dsg3 consists of 5 extracellular domains, two of which, namely EC1 and EC2, contain the immunodominant epitopes P1-P4 analyzed in this study in terms of their activating potency. Positions and aminoacidic sequences of P1-P4 are outlined in the **Figure 3A**. While the general trend in pemphigus patients was increased compared to HC, stimulation with P2 appeared to be the most potent (p=0.16; **Figure 3B**). Additionally, no statistical difference was found between the increase of CD154 expression induced by desmoglein 3 or P2_206-220_ (**Figure 3C**). Considering T cell activation critically depends on the recognition of epitopes of the Dsg3 ectodomain, T cell epitopes of Dsg3 (specifically P2) may be sufficient to induce a robust CD4+ T and B cell response *via* CD40-CD154 against human Dsg3 leading to the production of pathogenic Dsg3 IgG antibodies.

## Discussion

The CD40/CD40 ligand (CD40L; CD154) co-stimulatory system, which amplifies immune responses and potentially induces inflammation, is considered to have a prominent role in autoimmune skin manifestations (32). Specific T cell recognition is increasingly considered a key component in the disease control and maintaining efficient cellular immune responses. Detection and characterization of antigen-specific CD4+ T cells has extensively improved over the last years. Most applied techniques based on the incorporation of 3H-TdR or ELISPOT analysis, are, however, lengthy, single-cell-cytokine-driven or do not allow the assessment of which specific cell subpopulation is proliferating in response to Ag-specific stimulation (15–17). Current state-of-the-art technologies based on the usage of peptide-MHC multimers, such as dextramers, that directly assess antigen-specific Th cells and are not dependent on activation, may potentially miss functional T-cells that bear T-cell receptors (TCRs) with low affinity for a cognate antigen (33, 34). Moreover, the process of manufacturing particular reagents is costly and MHC alleles as well as immunodominant peptide epitopes must be defined in advance of manufacturing (35, 36). A potential improvement of the detection of autoreactive T cells can be achieved by *ex vivo* expansion prior to MHC multimer labeling, which on the downside, is very time-consuming. Therefore, the present study aimed at developing a specific, sensitive, and rapid method for the analysis of Dsg3-specific CD4+ T cells in whole PBMCs from PV patients by using CD154 activation marker. Unlike peptide-MHC multimers, CD154 *ex vivo* assay allows access to the whole population of CD4+ T cells specific for Dsg3, independent of MHC alleles or immunodominant peptide epitopes (20, 34).

Potent B cell activation followed by antibody production requires stimulation through the antigen receptor along with co-stimulation. CD40 receptor and its ligand CD154 (CD40L) are co-stimulatory molecules belonging to the TNFR superfamily. Binding of CD154 on CD4+ T cells to CD40 is a prerequisite for activation of B cells and other APCs (37, 38). T cell–dependent B cell activation is, in the immune pathogenesis of PV, critical for the induction of pathogenic IgG Abs, which directly induce epidermal loss of adhesion (12). Therefore, B cell depletion by the monoclonal anti-CD20 mAb rituximab in PV patients led to a decreased anti-Dsg3 IgG serum correlating with a marked downregulation of Dsg3-specific T cells (39). This strongly suggests that Dsg3-reactive T cells depend, to a great extent, on B cells as APCs (40, 41). Of note, when patients’ peripheral lymphocytes were depleted of CD4+ T cells, anti-Dsg3 IgG–producing B cells were no longer detectable. Two independent groups showed in PV animal models that a single Dsg3-reactive T cell clone was sufficient to prime naive B cells to produce Dsg3-specific pathogenic IgG autoantibodies (42, 43). Therefore, using an analytic tool to detect T lymphocyte proliferative responses is of great importance in PV for both diagnosis and monitoring of treatment response.

The present study analyzes the expression of *de novo* synthesized CD154 after short-term activation. Kinetic analysis of optimal duration of *ex vivo* stimulation was 16 hours, which corresponds to related studies (44). The transient nature of CD154 expression requires its intracellular stabilization using Brefeldin A, which blocks protein degradation by preventing transport processes during cell activation, allowing the combined identification of rare Dsg3-specific and cytokine producing T cells in PV (45). Subsequent analysis revealed that after antigen-specific T cell stimulation, CD154 was significantly increased in CD4+ T cells from PV patients compared to HC, and directly correlated to anti-Dsg3 Ab-titers. Studies on T cells show that T follicular helper (Tfh) cells are critical for initiating autoreactive B cell responses (46–48). Moreover, Dsg3-specific Th2 lymphocytes were found in different disease stages of PV disease (acute/clinically active, chronic and remittent (clinically healed) (49). Using CXCR5, marker for homing into follicles of secondary lymphoid organs, we found a significant induction only in acute patients in both Th and Tfh CD4+ subpopulations compared to remittent and HC. Additional analysis of co-expression of CD154 with cytokines allows more detailed cellular activation profiling. Previous studies of T cells in the skin of PV patients reported elevated concentrations of IL-4 and IFN-y as well as, more recently, IL-17A and IL-21 (50, 51). We found a significant induction of IL-17 and IL-21 in CD154+CD4+ T cells from PV patients compared to HC, meanwhile CD154- CD4+ T cells remained mainly unaffected regarding their cytokine expression after antigenic stimulation. This is in line with results provided by Holstein et al. showing that peripheral blood T cell subsets of patients with active pemphigus are dominated by IL-17-producing Th and Tfh cell subsets (7). IL-17A was previously found to play a significant role at epithelial barrier sites by inducing the expression of other proinflammatory cytokines and chemokines. Additionally, IL-17 improves epithelial infiltration and thereby pushing further ongoing inflammation of local autoimmune reactions, also by triggering a positive-feedback loop *via* an IL-6 induction (52). IL-21 not only induces B cell dependent antibody formation (53), but also promotes Th17 maturation in naïve CD4+ T cells (54). Therefore, co-expression of CD154 in CD4+ T cells with IL-17 and IL-21 only further emphasizes the concept of CD154 as a specific activation marker in PV.

Several studies have by now provided evidence that PV-associated HLA class II alleles are involved in the activation of Dsg3-reactive CD4+ T cells. Moreover, even CD4+ T cell responses against specific peptides of the Dsg3 ectodomain were identified in PV patients (11, 55) and HLA-DRB1*04:02–binding Dsg3 T cell epitopes in a corresponding mouse model (12). This prompted us to inquire whether stimulation with Dsg3 specific epitopes would suffice to induce CD154 expression in antigen-specific CD4+ T cells. According to the published sequence of Dsg3, position and amino acid sequences of used immuno-reactive epitopes (P1-P4) are outlined in the **Figure 3A**. Dsg3 peptide P2_(206-220)_ was found to induce CD4+ T cells most effectively in PV patients compared to HC and to the similar extend as the full Dsg3 protein. In *in silico* modeling, P2 was found to be capable of binding only to DRB1*0402 (56), however, a respective clone displays a strong PV characteristic polarization towards Th2 by the secretion of IL-4 and IL-10 after stimulation with Dsg3 (11). This further highlights that this epitope alone could suffice for the detection of Dsg3-specific CD4+ T cells in PBMCs of PV.

To this end, we developed a method in which CD4+ T cells from fresh PBMCs could be *ex vivo* stimulated with either whole protein antigen (Dsg3) or immunodominant peptides (P1-P4) and after stimulation analyzed for the activation marker CD154 (CD40L). The presented methodological approach could be used to further advance our knowledge about the actual frequency and characteristics of CD4+ T cell reactivity in PV patients. Positive IL-17 and IL-21 co-expression as well as correlation of anti-Dsg3 titers with CD154 expression in PV patients compared to healthy controls further supports the concept that immune activity in PV relies, at least partially, on Dsg3 reactive Th17 T cell subsets. These findings strongly suggest that CD154 as a specific activation marker in PV, expressed by antigen-specific CD4+ T cells, is critical during the pathogenesis of PV. Moreover, upregulation of IL-21 and IL-17 were seen to associate with antigen-specific activation, therefore lend themselves as potential therapeutic targets in pemphigus.

## Data availability statement

The original contributions presented in the study are included in the article/**Supplementary Material**. Further inquiries can be directed to the corresponding author.

## Ethics statement

The studies involving human participants were reviewed and approved by the Ethics Committees of the Medical Faculty of the Philipps-Universität Marburg (Az. 20/14) and the Charité Berlin (AZ: EA4/194/19) and was conducted according to the principles of the Declaration of Helsinki. The patients/participants provided their written informed consent to participate in this study.

## Author contributions

Conceptualization: CH. Data curation: AP, LK, and AI. Formal analysis: AP and CH. Funding acquisition: MH and CM. Investigation: CH, AP, LK, and AI. Project administration: CH. Supervision: CH and CM. Patient recruitment: DD and FS. Writing original draft: AP and CH. Writing – review & editing: AP, LK, AI, MH, CM, CH, DD, FS, and KG. All authors read and approved the final version of the manuscript.

## Funding

This work was supported by a grant from the Deutsche Forschungsgemeinschaft (DFG) FOR 2497 Pegasus to MH (TP08; HE 1602/16-2), CM (TP02; MO 2076/4-2) and KG (TP02; GH133/2-2) to KG. FS is a participant in the BIH Charité Clinician Scientist Program funded by the Charité – Universitätsmedizin Berlin, and the Berlin Institute of Health at Charité (BIH). Open Access funding provided to CH by the Open Access Publication Fund of Philipps-Universität Marburg with support of the Deutsche Forschungsgemeinschaft (DFG, German Research Foundation).

## Acknowledgments

We thank Manuel Schulze-Dasbeck and Nicole Löwer for excellent technical help. Determination of HLA class was performed by the staff of the University Hospital Giessen.

## Conflict of interest

MH is a recipient of an unrestricted grant from TOPAS Therapeutics relating to a collaboration aimed at inducing therapeutic T cell tolerance in pemphigus. 

The remaining authors declare that the research was conducted in the absence of any commercial or financial relationships that could be construed as a potential conflict of interest.

## Publisher’s note

All claims expressed in this article are solely those of the authors and do not necessarily represent those of their affiliated organizations, or those of the publisher, the editors and the reviewers. Any product that may be evaluated in this article, or claim that may be made by its manufacturer, is not guaranteed or endorsed by the publisher.
