# Supplementary material for: Detection of rare autoreactive T cell subsets in patients with pemphigus vulgaris
Source: Front Immunol. 2022 Sep 20;13:979277. doi: 10.3389/fimmu.2022.979277 (PMC9531257; doi:10.3389/fimmu.2022.979277)
Supplement: Supplementary file 1 [file DataSheet_1.docx]

1. Detection of rare autoreactive T cell subsets in patients with pemphigus vulgaris

**Short title:** Detection of desmoglein 3 reactive T cells

Polakova A.^1#^, Kauter L.^1#^, Ismagabetova A.^1^, Didona D.^1^, Solimani F.^2,3^, Ghoreschi K.^2^, Hertl M.^1^, Möbs C.^1^, Hudemann C.^1*^

^1^Department of Dermatology and Allergology, Philipps-Universität Marburg, Marburg, Germany

^2^ Department of Dermatology, Venereology and Allergology, Charité-Universitätsmedizin Berlin, Berlin, Germany

^3^ Berlin Institute of Health at Charité – Universitätsmedizin Berlin, BIH Biomedical Innovation Academy, BIH Charité Clinician Scientist Program, Charitéplatz 1, 10117 Berlin, Germany

- **Supplementary -**

*** Correspondence to:**

Christoph Hudemann, PhD

[christoph.hudemann@staff.uni-marburg.de](mailto:christoph.hudemann@staff.uni-marburg.de)

*Keywords*: CD154, CD40L, CD4+ T cells, pemphigus, autoreactive, desmoglein, autoimmunity

**Supplementary Tables and Figures**


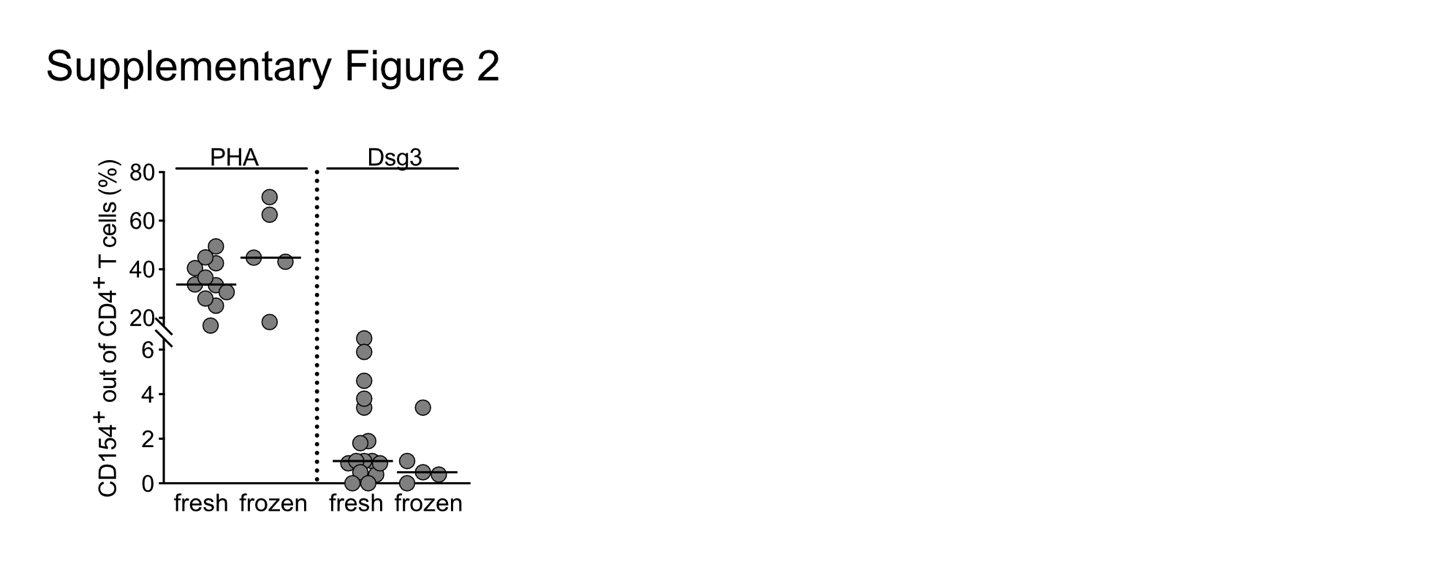


**Supplementary Figure 1. Expression of CD154 upon specific (Dsg3) and nonspecific (PHA) stimulation of fresh and frozen PBMCs.** PBMCs from PV patients processed either directly after withdrawal (fresh) or stored frozen in liquid nitrogen (frozen) were stimulated specifically (Dsg3) or polyclonally (PHA) and relative frequencies of CD154+ out of CD4+ T cells were plotted (fresh n=11; frozen n=5).

| **Patient** | **Sex** | **Age** | **Status** | **Therapy** | **RTX** | **Dsg1/Dsg3 [RE/ml]** | **Clinical phenotype** | **HLA-type** |
| --- | --- | --- | --- | --- | --- | --- | --- | --- |
| 01 | f | 67 | Remission | - | - | 6/161 | mucosal | HR DRβ1* 0402 |
| 02 | f | 41 | Remission | 150mg Aza | - | 4/141 | - | n/a |
| 03 | f | 45 | Remission | 100mg Aza | - | 0/564 | mucocuta-neos | HR DRβ1* 0402,  HR DQβ1* 0503 |
| 04 | m | 52 | Remission | 7,5mg Pred,  200mg Aza | 5 | 0/4 | - | HR DRβ1* 0402 |
| 05 | m | 60 | Remission | 5mg Pred | - | 3/10 | mucosal | HR DQβ1* 0503 |
| 06 | f | 45 | Remission | 7,5mg Pred | - | 978/15 | cutaneous | HR DQβ1* 0503 |
| 07 | f | 45 | Remission | 150mg Aza,  7mg Pred | 25 | 3/607 | mucosal | HR DRβ1* 0402,  HR DQβ1* 0503 |
| 08 | f | 55 | Remission | 5mg Pred, MMF | 7 | 56/161 | mucocuta-neos | HR DQβ1* 0503 |
| 09 | f | 61 | Remission | - | - | 12/291 | - | HR DQβ1* 0503 |
| 10 | m | 81 | Remission | - | - | 5/199 | - | not PV specific |
| 11 | m | 67 | Remission | - | - | 9/132 | - | HR DRβ1* 0402,  HR DQβ1* 0503 |
| 12 | f | 65 | Remission | - | 2 | 0/111 | mucosal | HR DRβ1* 0402 |
| 13 | f | 61 | Remission | 100mg Aza | - | 0/16 | - | HR DRβ1* 0402 |
| 14 | f | 64 | Remission | 30mg Pred | 12 | 15/20 | - | HR DRβ1* 0402 |
| 15 | f | 60 | Remission | 100mg Aza,  20mg Pred | - | 19/51 | cutaneous | HR DQβ1* 0503 |
| 16 | f | 54 | Remission | Rituximab, 1000mg Aza, 1000mg Pred | 0 | 2/20 | mucocuta-neos | HR DQβ1* 0503 |
| 17 | m | 62 | Acute | 100mg Aza, 20mg Pred | - | 408/814 | cutaneous | n/a |
| 18 | f | 44 | Acute | 40mg Pred | - | 18/79 | n/a | n/a |
| 19 | f | 62 | Acute | Dapson 100mg, 7,5mg Pred | - | 2/52 | n/a | n/a |
| 20 | m | 83 | Acute | MMF | - | 136/565 | mucocuta-neos | n/a |
| 21 | f | 43 | Acute | - | - | 2/66 | n/a | n/a |
| 22 | m | 61 | Remission | - | - | 05/49 | mucosal | n/a |
| 23 | m | 49 | Acute | 100mg Aza, 20mg Pred | - | 9/275 | mucosal | n/a |
| 24 | m | 58 | Acute | 10mg Pred | 9 | 131/16 | mucocuta-neos | HR DRβ1* 0402, HR DQβ1* 0503 |
| 25 | f | 60 | Acute | Dexamethason-Pulstherapy, IVIg 140g | - | 6/183 | mucocuta-neos | n/a |
| 26 | m | 47 | Acute | - | - | 70/145 | mucocuta-neos | n/a |
| 27 | f | 46 | Remission | 150mg Aza, 7mg Pred | 32 | 16/911 | mucosal | HR DRβ1* 0402, HR DQβ1* 0503 |
| 28 | m | 49 | Remission | - | - | 212/129 | mucocuta-neos | n/a |
| 29 | f | 83 | Remission | - | - | 0/3 | - | n/a |
| 30 | f | 73 | Remission | 50mg Pred | - | 157/3 | cutaneous | n/a |
| 31 | f | 54 | Remission | 5mg Pred | - | 3/3 | mucosal | - |
| 32 | m | 83 | Remission | - | - | 0/165 | - | HR DQβ1* 0503 |
| 33 | m | 44 | Remission | MMF, 20mg Pred | - | 0/18 | mucosal | n/a |

**Supplementary Table 1. Epidemiological data, clinical status, and clinical phenotype, Dsg titer, HLA type of pemphigus patients.** Clinical status of patients, i.e., acute or remission, was defined based on Murell et al. 2008. Daily doses of indicated therapy until the point of collection of peripheral blood for analysis are outlined under therapy (Pred Prednisolone; Aza Azathioprine; MMF Mycophenolat-Mofetil; n/a not available). Months after last dosis of B cell depletion therapy with Rituximab (RTX; anti-CD20 antibody). Dsg1 and Dsg3 titers were determined by ELISA (EUROIMMUN) with recombinant Dsg1/Dsg3 [relative units/ml], where limit value is 20 RE/ml. PV specific HLA class II haplotypes are HR DRβ1*0402, HR DQβ1*0503. N/a data not available.

| **Healthy control** | **Sex** | **Age** |
| --- | --- | --- |
| 01 | f | 24 |
| 02 | m | 24 |
| 03 | m | 41 |
| 04 | m | 30 |
| 05 | m | 23 |
| 06 | m | 41 |
| 07 | f | 23 |
| 08 | m | 32 |
| 09 | f | 22 |
| 11 | f | 28 |
| 12 | m | 26 |
| 13 | m | 47 |
| 14 | f | 34 |
| 15 | m | 27 |
| 16 | m | 41 |
| 17 | m | 36 |
| 18 | f | 58 |
| 19 | f | 39 |
| 20 | m | 54 |
| 21 | m | 51 |
| 22 | f | 32 |
| 23 | m | 33 |

**Supplementary Table 2. Epidemiological data of healthy controls.**

| **Figure 1** | **C** | median CD154+ out of | | | | | |
| --- | --- | --- | --- | --- | --- | --- | --- |
|  |  |  | 2h | 6h | 12h | 16h | 22h |
|  |  | **CD3+T cells** | 7,33 | 19,47 | 34,79 | 33,88 | 26,91 |
|  |  | **CD3+CD4- T cells** | 0,11 | 4,9 | 6,72 | 3,37 | 3,31 |
|  |  | **CD3+CD4+ T cells** | 15,39 | 34,32 | 56,17 | 57,62 | 44,56 |
|  |  | **CD3+CD4+CXCR5- T cells** | 11,48 | 23,64 | 39,84 | 41,15 | 34,15 |
|  |  | **CD3+CD4+CXCR5+ T cells** | 10,44 | 25,36 | 53,72 | 63,74 | 42,54 |
|  | **D** | median | | | | | |
|  |  | CD154+ out of **CD8+ Dsg3** | | | | | 0,1 |
|  |  | CD154+ out of **CD8+ PHA** | | | | | 0,2 |
|  |  | CD154+ out of **CD4+ Dsg3** | | | | | 1,8 |
|  |  | CD154+ out of **CD4+ PHA** | | | | | 33,8 |
| **Figure 2** | **A** | median | | | | | |
|  |  | **PV** (**Dsg3-reactive** CD154+ out of **CD4+ T cells**) | | | | | 0,24 |
|  |  | **HC** (**Dsg3-reactive** CD154+ out of **CD4+ T cells**) | | | | | 0 |
|  | **D** | median | | | | | |
|  |  | **PV IL-4+** (Dsg3-reactive **CD154+CD4+ T cells**) | | | | | 0,130 |
|  |  | **HC IL-4+** (Dsg3-reactive **CD154+CD4+ T cells**) | | | | | 0,045 |
|  |  | **PV IL-17+** (Dsg3-reactive **CD154+CD4+ T cells**) | | | | | 0,475 |
|  |  | **HC IL-17+** (Dsg3-reactive **CD154+CD4+ T cells**) | | | | | 0,005 |
|  |  | **PV IL-21+** (Dsg3-reactive **CD154+CD4+ T cells**) | | | | | 0,330 |
|  |  | **HC IL-21+** (Dsg3-reactive **CD154+CD4+ T cells**) | | | | | 0,000 |
|  |  | **PV IFN-γ+** (Dsg3-reactive **CD154+CD4+ T cells**) | | | | | 0,006 |
|  |  | **HC IFN-γ+** (Dsg3-reactive **CD154+CD4+ T cells**) | | | | | 0,045 |
|  |  | **PV IL-4+** (**CD154-CD4+ T cells**) | | | | | 0,020 |
|  |  | **HC IL-4+** (**CD154-CD4+ T cells)** | | | | | 0,024 |
|  |  | **PV IL-17+** (**CD154-CD4+ T cells**) | | | | | 0,017 |
|  |  | **HC IL-17+** (**CD154-CD4+ T cells**) | | | | | 0,007 |
|  |  | **PV IL-21+** (**CD154-CD4+ T cells**) | | | | | 0,000 |
|  |  | **HC IL-21+** (**CD154-CD4+ T cells**) | | | | | 0,000 |
|  |  | **PV IFN-γ+** (**CD154-CD4+ T cells**) | | | | | 0,000 |
|  |  | **HC IFN-γ+** (**CD154-CD4+ T cells**) | | | | | 0,025 |
|  | **F** | median | | | | | |
|  |  | **acute PV** (**Dsg3-reactive** CD154+ out of **CXCR5-CD4+ T cells**) | | | | | 0,47 |
|  |  | **remittent PV** (**Dsg3-reactive** CD154+ out of **CXCR5-CD4+ T cells**) | | | | | 0,05 |
|  |  | **HC** (**Dsg3-reactive** CD154+ out of **CXCR5-CD4+ T cells**) | | | | | 0 |
|  |  | **acute PV** (**Dsg3-reactive** CD154+ out of **CXCR5+CD4+ T cells**) | | | | | 0,55 |
|  |  | **remittent PV** (**Dsg3-reactive** CD154+ out of **CXCR5+CD4+ T cells**) | | | | | 0,02 |
|  |  | **HC** (**Dsg3-reactive** CD154+ out of **CXCR5+CD4+ T cells**) | | | | | 0 |
| **Figure 3** | **B** | median | | | | | |
|  |  | **PV P1** (CD154+ out of CD4+ T cells) | | | | | 0,6 |
|  |  | **PV P2** (CD154+ out of CD4+ T cells) | | | | | 1,7 |
|  |  | **PV P3** (CD154+ out of CD4+ T cells) | | | | | 0,5 |
|  |  | **PV P4** (CD154+ out of CD4+ T cells) | | | | | 0 |
|  |  | **HC P1** (CD154+ out of CD4+ T cells) | | | | | 0 |
|  |  | **HC P2** (CD154+ out of CD4+ T cells) | | | | | 0 |
|  |  | **HC P3** (CD154+ out of CD4+ T cells) | | | | | 0 |
|  |  | **HC P4** (CD154+ out of CD4+ T cells) | | | | | 0 |
|  | **C** | median | | | | | |
|  |  | **PV Dsg3** (CD154+ out of CD4+ T cells) | | | | | 1,8 |
|  |  | **PV P2** (CD154+ out of CD4+ T cells) | | | | | 1,7 |
| **Suppl. Figure 1** |  | median | | | | | |
|  |  | **fresh PHA** (CD154+ out of CD4+ T cells) | | | | | 33,8 |
|  |  | **frozen PHA** (CD154+ out of CD4+ T cells) | | | | | 44,8 |
|  |  | **fresh Dsg3** (CD154+ out of CD4+ T cells) | | | | | 1 |
|  |  | **frozen Dsg3** (CD154+ out of CD4+ T cells) | | | | | 0,5 |

**Supplementary Table 3. Median values of data presented in respective figures.**
